# Automated data analysis to rapidly derive and communicate ecological insights from satellite-tag data: A case study of reintroduced red kites

**DOI:** 10.1007/s13280-015-0711-3

**Published:** 2015-10-27

**Authors:** René van der Wal, Cheng Zeng, Danny Heptinstall, Kapila Ponnamperuma, Chris Mellish, Stuart Ben, Advaith Siddharthan

**Affiliations:** Aberdeen Centre for Environmental Sustainability, School of Biological Sciences, University of Aberdeen, Aberdeen, AB24 3UU UK; Computing Science, University of Aberdeen, Aberdeen, AB24 3UE UK; ARRIA R&D, University of Aberdeen, Meston Building G05E, Aberdeen, AB24 3UE UK; RSPB North Scotland, Etive House, Beechwood Park, Inverness, IV2 3BW UK

**Keywords:** Automated home-range calculation, Red kite reintroduction, Satellite-tag data, Science communication

## Abstract

Analysis of satellite-telemetry data mostly occurs long after it has been collected, due to the time and effort needed to collate and interpret such material. Delayed reporting reduces the usefulness of such data for nature conservation where timely information about animal movements is required. To counter this problem, we present a novel approach which combines automated analysis of satellite-telemetry data with rapid communication of insights derived from such data. A relatively simple algorithm (based on radial and angular velocity calculated from fixes) allowed instantaneous detection of excursions away from settlement areas and automated calculation of home ranges on the remaining data. Automating the detection of both excursions and home-range calculations enabled us to disseminate ecological insights from satellite-tag data instantaneously through a dedicated web portal. The automated analysis, interpretation, and communication of satellite-tag and other ecological data offer clear benefits to nature conservation research and practice.

**Electronic supplementary material:**

The online version of this article (doi:10.1007/s13280-015-0711-3) contains supplementary material, which is available to authorized users.

## Introduction

The management and conservation of animal populations require an understanding of the spatial use of individuals over time (Chetkiewicz et al. 2006; Cooke 2008). Technological advances have greatly improved the efficiency with which such ‘animal movement data’ can be gathered; this has most notably been achieved through the use of Global Navigation Satellite Systems (GNSS), such as the American Global Positioning System (GPS) (Tomkiewicz et al. 2010). Although its origins are in military aircraft location, miniaturisation as well as increased energy efficiency and cost reductions have resulted in new GPS chips which are available for a wide variety of applications, including animal tracking (Curatolo and Cornelius 2012).

Indeed, satellite tags are rapidly replacing traditional radio-based technologies for animal tracking as larger quantities of considerably more accurate data can be gathered with far greater ease (Girard et al. 2002; Tomkiewicz et al. 2010). Drivers behind the increasing popularity of GPS tags include: further miniaturisation (allowing their use on ever smaller animals); reduced cost (allowing wider uptake and inclusion in studies that require larger numbers of tagged individuals); and the incorporation of additional sensors to allow on-animal monitoring of external variables (such as daylight, diving depth, or air temperature) and internal variables (such as heart rate) to enable the answering of additional research questions. Many recent satellite-telemetry studies, and methodological advances therein (e.g. Aarts et al. 2008), originate from the marine realm (notably concerning animals that breach the water surface). This suggests that satellite telemetry is being used for investigations which could not have previously been undertaken with traditional radio-based technologies (e.g. due to the limited utility of short-range radio signals in vast oceans; see Cooke et al. 2013 for a review).

The application of satellite telemetry in nature conservation is now commonplace, with animal movement data improving the evidence base managers and policymakers can draw upon. Topics are wide ranging and include identification of migration routes, existence of habitat corridors, use of protected areas, locations of individual mortality, and managing animal movement through ‘geo-fencing’ (Girard et al. 2002; Krapu et al. 2011; Wall et al. 2014). Additionally, satellite telemetry is being used as a public engagement tool at both local (Witt et al. 2014,1) and national scales.^2^

Although the use of GPS telemetry offers many advantages, analysis mostly occurs long after the data has been gathered due to the amount of time needed to handle and interpret the often large quantities of data concerned (Hebblewhite and Haydon 2010). While delayed reporting on findings may not necessarily constrain ecological research, it does reduce the utility of GPS telemetry in nature conservation where immediate analysis and interpretation of data are needed to guide action (Wall et al. 2014), or where the real-time communication of animal movements is used to engage wider audiences (Verma et al. 2015). Here we present an approach which allows real-time analysis of GPS-telemetry data, resulting in ecological insights which can directly be visualised and communicated to various audiences. For this, we centred on home ranges as one of the central concepts of the animal movement research field.

The calculation of animal home ranges, that is, the delineation of areas of intensive use by individuals, is frequently undertaken by ecologists and conservation biologists as a means to better understand or predict spatial–temporal patterns of habitat or landscape use (Kie et al. 2010). Such analyses can improve our fundamental understanding of resource utilisation and inter/intra-specific interactions, as well as help inform management decisions. Given that the term ‘home range’ typically captures ‘where individuals are found for most of the time’, they are often reported for situations in which individuals have to remain faithful to a specific area (e.g. because of reproductive commitments). During other parts of an animals’ life cycle, however, ties to a specific area may be less. For example, during the transience phase of dispersal (Clobert et al. 2009), as part of the prospecting process, individuals may intensively use an area for a discrete time period before moving into another area of intensive use. These transiently used areas are often referred to as ‘temporary settlement areas’ in order to distinguish them from areas of intensive use where an individual is obliged to return to the area (e.g. a home range around a breeding site) (del Mar Delgado and Penteriani 2008).

Although many methods to calculate home ranges exist, most are based on the principle of excluding a certain percentage of observations (i.e. location fixes) to derive at a sufficiently narrow delineation of a ‘home range’ (e.g. 90 or 95 % minimum convex polygons or kernels) (Kie et al. 2010). These methods exclude outlying data in order to derive at an area in which an individual is most likely to be found; the same principle applies when home ranges are derived on the basis of location fix densities (Keating and Cherry 2009). However, any such routines ignore the fact that individuals of many species regularly undertake excursions away from their home range (e.g. del Mar Delgado et al. 2010; Carlson et al. 2014). Because such movements are often distinct and conceptually different to movements undertaken within a home range, it seems appropriate to exclude movement data relating to excursions when calculating a home range. That way, home ranges would be derived on fixes obtained from individuals expressing behaviours in line with the home-range concept.

In this study, we present a simple method to automatically detect and exclude excursion data so that home ranges may be automatically calculated on the remaining data, and we compare how our estimates of home ranges differ from those calculated by traditional methods. Moreover, we demonstrate how automated identification of home ranges and excursions enables instantaneous dissemination of ecological insights from satellite-tag data through a dedicated web portal to inform conservationists and members of the public.

## Materials and methods

### Case study

The study focuses on a population of satellite-tagged red kites (*Milvus milvus*) on the Black Isle near Inverness in the north of Scotland. Historically common across the Great Britain, red kites were considered benign until the eighteenth century when they became perceived as a threat to game and farming interests; the resulting systematic killing (Lovegrove 2007) reduced the number of successfully breeding red kites to less than 10 pairs by the mid-twentieth century (Davis 1993). In order to restore red kites to their previously occupied breeding range across the UK, conservationists reintroduced them to 10 locations in the late twentieth century (Carter 2007). As attitudes toward red kites had softened among many in the game and farming industries, these reintroductions were mostly successful. However, our focal population in the north of Scotland continued to experience high rates of mortality (primarily due to illegal poisoning, be it passive or otherwise), and consequently exhibited very low population growth rates (Smart et al. 2010).

In an effort to turn around the fate of this local population, conservationists fitted satellite tags (PTT-100 22 gram Solar Argos/GPS PTT^3^) to red kite chicks immediately prior to fledging, consequently capturing the movements of red kites during the transience phase of post-natal dispersal. The tags were solar-powered and programmed to transmit up to six location fixes a day. Although up to six fixes could indeed be acquired during the summer months, a lack of sunlight in Scotland meant fewer fixes (a maximum of four per day) could be obtained in spring and autumn, and only the occasional one during winter. The conservation charity in charge of the reintroduction (the Royal Society for the Protection of Birds, RSPB) had employed these tags for two reasons, namely to foster the detection and prosecution of wildlife crime, and to provide real-time data on the movements of individual red kites (often adopted and named by local schools) to assist in engaging the local community with the reintroduction programme and the species’ wider conservation needs. An ability to rapidly understand, interpret and communicate the movement patterns of dispersing red kites were therefore required to meet these aims.

**Fig. 1:**
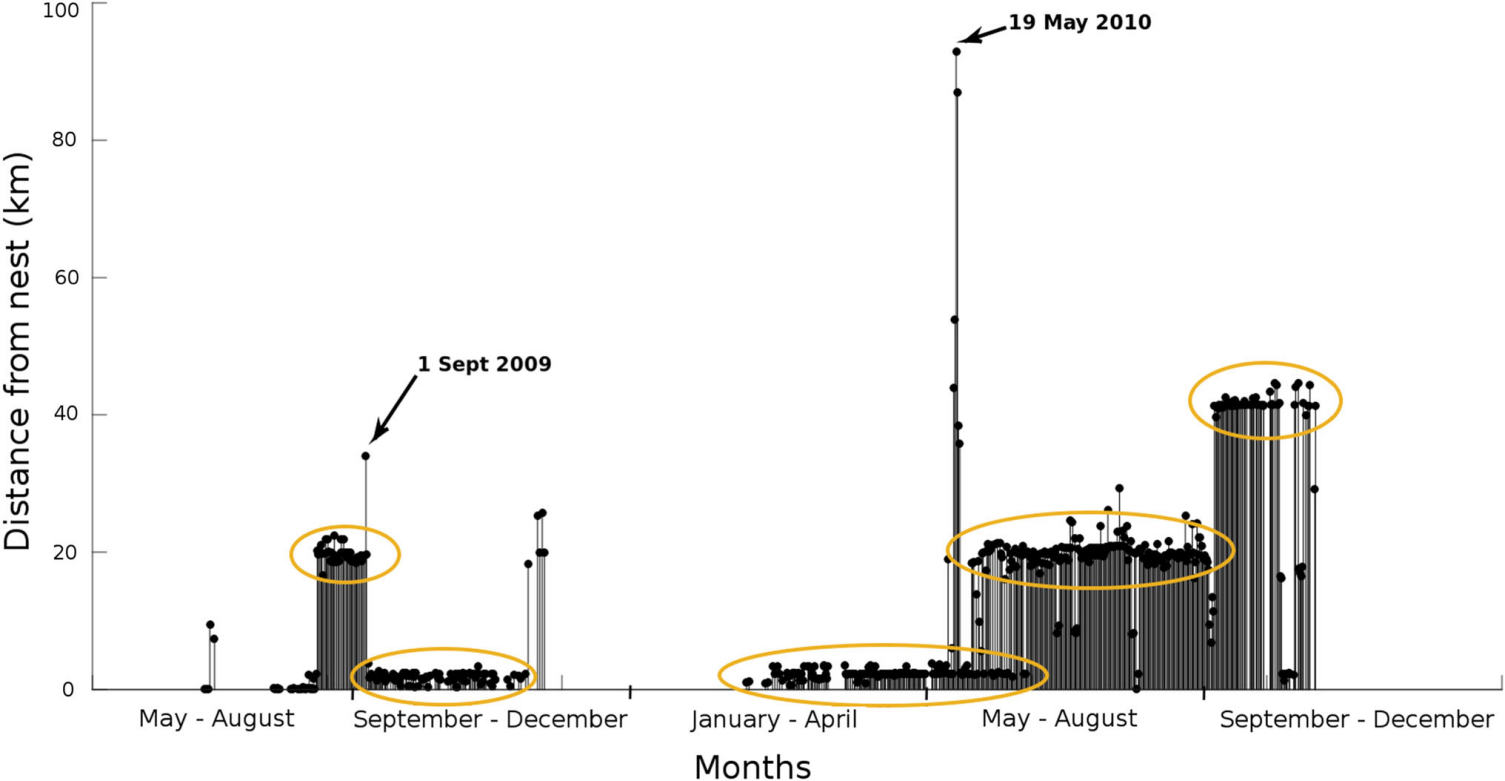
Representation of movement data for one satellite-tagged red kite (bird 93933b) from the Black Isle, NE Scotland over the period May 2009 to December 2010. Plotted is the distance (in km) from the individual’s natal site (all tags were placed on chicks in the nest) over time. *Amber ellipses* indicate time periods during which the bird has settled in a given area (temporary settlement areas). *Arrows* point at two clear explorations undertaken before using a new temporary settlement area. Absence of *bars* indicate days without data, a situation primarily occurring during winter when the solar-powered tags have too low an energy status for communication with a satellite

**Fig. 2:**
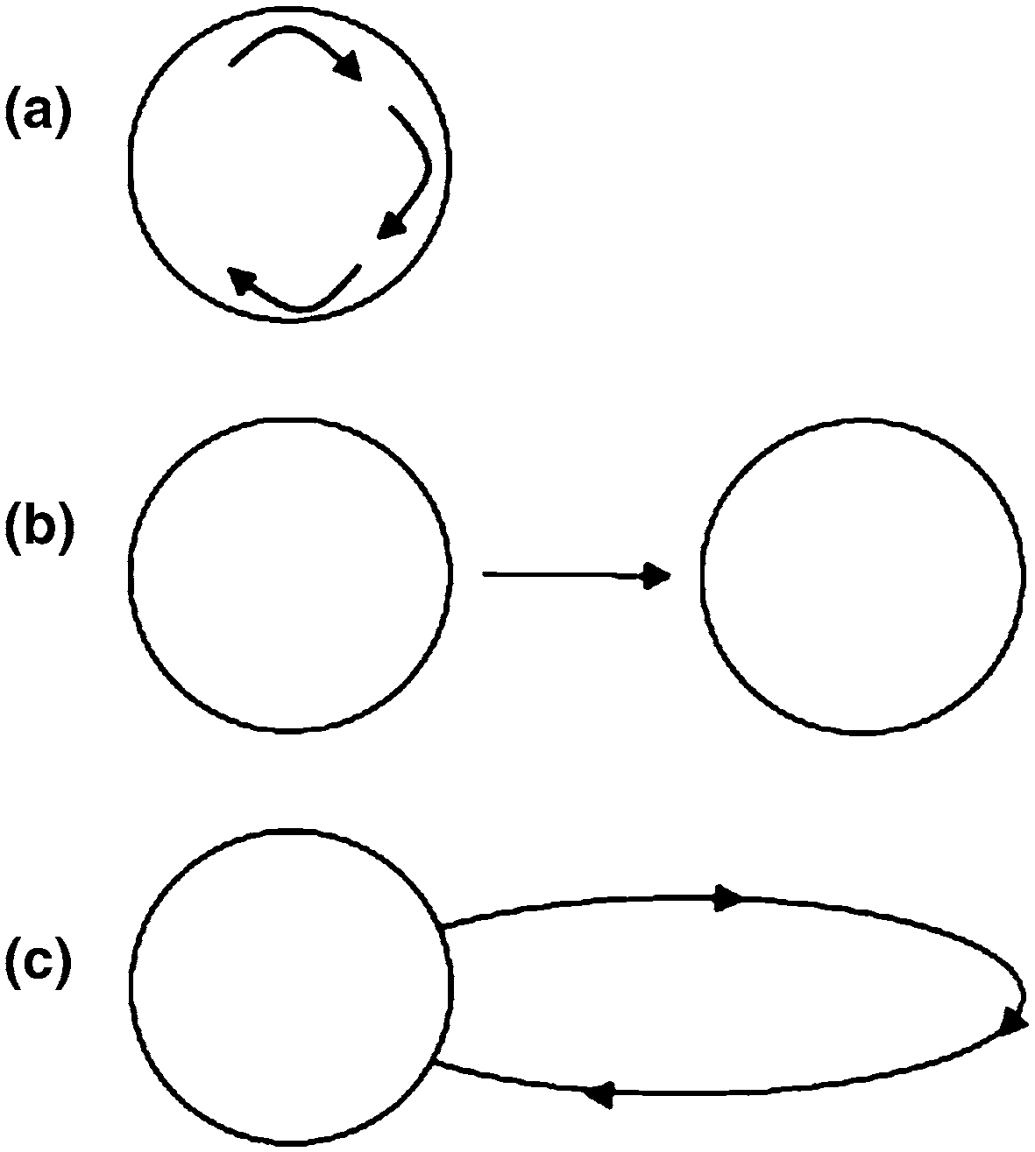
Diagrammatic representations of three red kite movement patterns identified from satellite-tag data: **a** small and constricted movements within an area of intense use (i.e. a home range or temporary settlement area); **b** direct movements between separate areas of intense use; and **c** exploratory movement from an area of intense use (round trip)

Satellite-telemetry data were received from 24 individuals over different periods and lengths of time (Table 1). Particularly for these reasons, but also due to the previously discussed seasonal fluctuation in solar energy, there were large differences in the total number of fixes obtained among individual red kites (Table 1). For some birds the study period was so short that most movement was very local (near the nest site); however, most birds were followed for much longer periods during which movement often took place over larger spatial scales (much of Scotland and parts of England). Initial data exploration was achieved by automatic processing and plotting of the data onto Google Maps (Ponnamperuma et al. 2013). This functionality revealed the first detailed data on movement patterns of red kites during the transience phase of post-natal dispersal; it demonstrated that their prospecting behaviour is composed of shorter and longer excursions interspersed with intensive use of specific areas (i.e. temporary settlement areas) for varying lengths of time (Figs. 1, 2). This finding confirmed that it would not be appropriate to calculate standard home ranges using traditional techniques, unless excursion data could be identified and excluded.

**Table 1:** Summary data of satellite-tagged red kites that were part of this study. For each individual kite (Bird ID), the study period and duration thereof (in months) is given alongside the total number of location fixes obtained during that period of time, and how many of those (and what percentage of total) were categorised by our algorithm as forming part of identified excursions

Bird ID	Study period (from/to)		Duration (months)	Total number of fixes	Number of excursion fixes	Percentage of excursion fixes
118721	21/10/2013	22/07/2014	9	229	46	20.1
113995	29/06/2012	25/10/2013	16	1594	181	11.4
93834	23/06/2009	13/09/2010	15	1494	149	10.0
54370	26/05/2010	08/11/2010	6	343	34	9.9
93833a	22/06/2012	21/11/2013	17	1694	162	9.6
83675	16/06/2009	30/04/2013	46	1995	135	6.8
93831	21/06/2009	14/11/2009	5	345	23	6.7
129013	16/07/2013	25/07/2013	1	48	3	6.3
84169	19/06/2012	28/09/2014	27	795	46	5.8
83673	15/06/2009	03/07/2010	13	1313	59	4.5
113994	27/06/2012	27/06/2014	24	1227	53	4.3
54372	19/06/2012	13/10/2014	28	2340	99	4.2
113993	25/06/2012	28/09/2014	29	1901	59	3.1
93835	03/07/2009	27/08/2014	61	2664	76	2.9
83674	16/06/2009	07/11/2010	17	1468	39	2.7
84170	20/06/2012	18/10/2014	28	1904	39	2.0
54373	26/05/2010	25/10/2010	5	303	5	1.7
93933b	23/06/2009	09/10/2010	16	1482	23	1.6
83671	31/07/2009	07/03/2010	9	280	4	1.4
93832	20/06/2009	15/04/2010	10	525	2	0.4
54374	26/05/2010	29/10/2010	5	229	0	0
54349	26/05/2010	07/11/2010	6	287	0	0
54344	26/05/2010	29/10/2010	5	277	0	0
83676	20/07/2009	25/09/2009	2	341	0	0

Thus, motivated by the need to identify excursions, remove such prospecting behaviour to calculate home ranges more adequately, and automate these procedures to allow rapid communication of the resulting ecological insights to conservation managers and members of the public, we set the following aims: (1) examine whether fixes belonging to excursions can be identified by a simple algorithm; (2) determine whether the exclusion of fixes identified as belonging to excursions will benefit home-range computations; and (3) determine whether identification of excursions and home ranges, if automated, allows for instantaneous interpretation of such data that can be communicated in real time to a wider audience.

### Data analysis

**Fig. 3:**
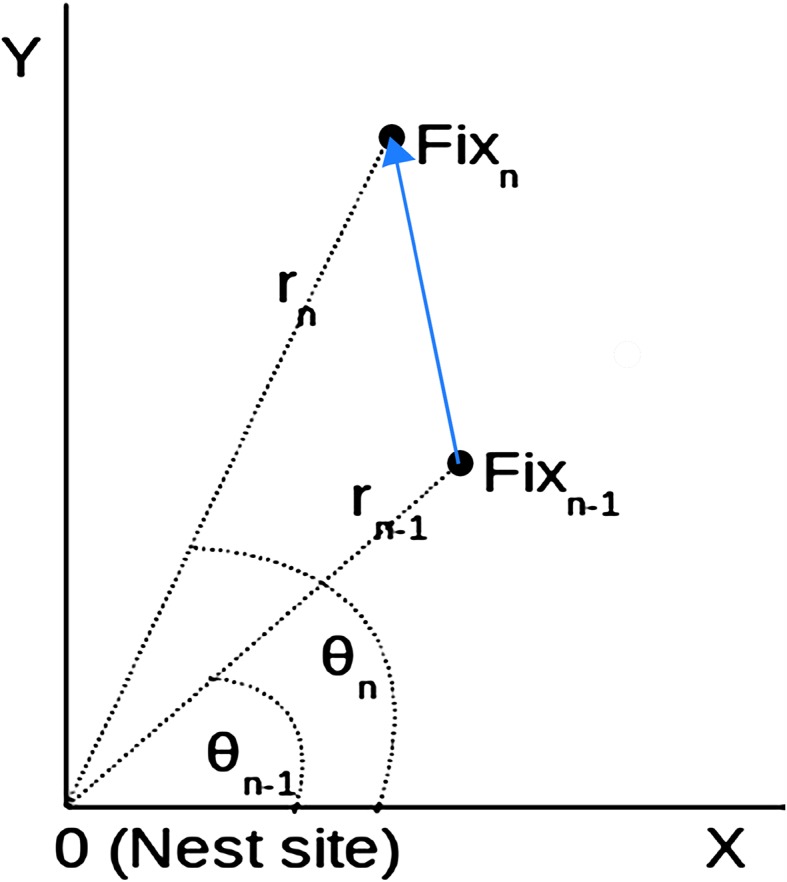
Polar coordinate system. Each location fix was represented as by a radius (distance from nest) and angle relative to the X axis. Radial and angular velocities capture how these coordinates change over time. Radial velocity captures speed of movement away from or toward the nest, and angular velocity captures speed of circular movement around the nest site

Initial data exploration (see Fig. 1) suggested that excursion behaviour manifests itself as relatively swift and straight movements, i.e. as movements with a relatively high radial velocity and low angular velocity. This behaviour contrasted with the limited extent of movement and wide range of directions (i.e. high angular velocity and low radial velocity) observed for individuals within areas of intense use (i.e. temporary settlement areas). To identify excursions (aim 1), we calculated angular and radial velocity in a polar coordinates frame (cf. Fig. 3). This required pre-processing of our GPS fixes. Location stamps were converted from latitude/longitude to x/y coordinates using the Lambert equal area projection (Snyder 1987) rescaled to kilometres; these, in turn, were converted to polar coordinates (*r*, *θ*) and centred on the nest location (because the first fixes of all kites were obtained from within the nest). Time stamps were converted from date/time to hours since the first fix. From this representation we calculated the following velocities:

1. radial velocity (i.e. speed away from or toward nest) *v*_r_ = *d*_r_/*d*_*t*_, the rate of change of radial coordinate, calculated for the *n*th fix as *v*_*r*_ = (*r*_*n*_ − *r*_*n*−1_)/(*t*_*n*_ − *t*_*n*−1_); and
2. angular velocity (i.e. speed around nest) *v*_*θ*_ = *d*_*θ*_/*d*_*t*_, the rate of change of angular coordinate, calculated for the *n*th fix as *v*_*θ*_ = (*θ*_*n*_ − *θ*_*n−*1_)/(*t*_*n*_ − *t*_*n*−1_).

The combination of large radial velocity* v*_r_ with small angular velocity* v*_θ_ represents directional movement, i.e. a relatively swift and direct travel from one point to the next. We classified points as being part of an excursion by applying the following heuristics:

1. Fixes within 30 km of the nest were not considered part of an excursion (to prevent misidentification of small-scale movement around the nest site).
2. Of all remaining ones, we considered fixes part of an excursion if radial velocity was high and angular velocity low. For this paper, we used values of *v*_r_ > 5 km/h and *v*_*θ*_ < 45°/h based on initial data exploration (see Supplementary material, Fig. 10.1007/s13280-015-0711-3 for the distribution of all angular velocity data).
3. Fixes were considered part of an excursion if they were more than 3 km away from any previous fix (i.e. the bird was further than 3 km from any previously known location and more than 30 km from the nest).

The rules above identified fixes to be part of an excursion based on behaviour exhibited locally. However, given the sparseness of the data (maximally six fixes per day), fixes which were part of a longer excursion ought to be labelled as such even if not locally exhibiting characteristics of an excursion. We therefore identified sequences of one, two, or maximally three fixes which lay between two excursion fixes, and considered these to be part of the same excursion (by relabelling the whole sequence as an excursion).

To determine whether exclusion of fixes identified as belonging to an excursion would influence home-range shape and position (aim 2), home ranges were calculated with and without excursion fixes. Home ranges were estimated by clustering the known locations of an individual over a period of time (Table 1) using 90 % kernels. For this, we made use of the adehabitat package for R (Calenge 2006); specifically, its implementation of a more recent algorithm for the estimation of the utilisation density in time and space using the product kernel estimator (Keating and Cherry 2009). Appropriate values for grid size and smoothing parameter were determined iteratively, and set as 90 and 0.04 respectively, to prevent too large a utilisation distribution with excess space around the outermost points within polygons (cf. Kie et al. 2010).

To determine whether the procedures that led to identified excursions and home ranges could facilitate instant interpretation (aim 3), rules were drawn up to identify specific behaviours; these were subsequently communicated through live blogs which were written by a computer using Natural Language Generation (see Ponnamperuma et al. (2013) for the infrastructure behind this innovation).

## Results

### Identification of excursions

**Fig. 4:**
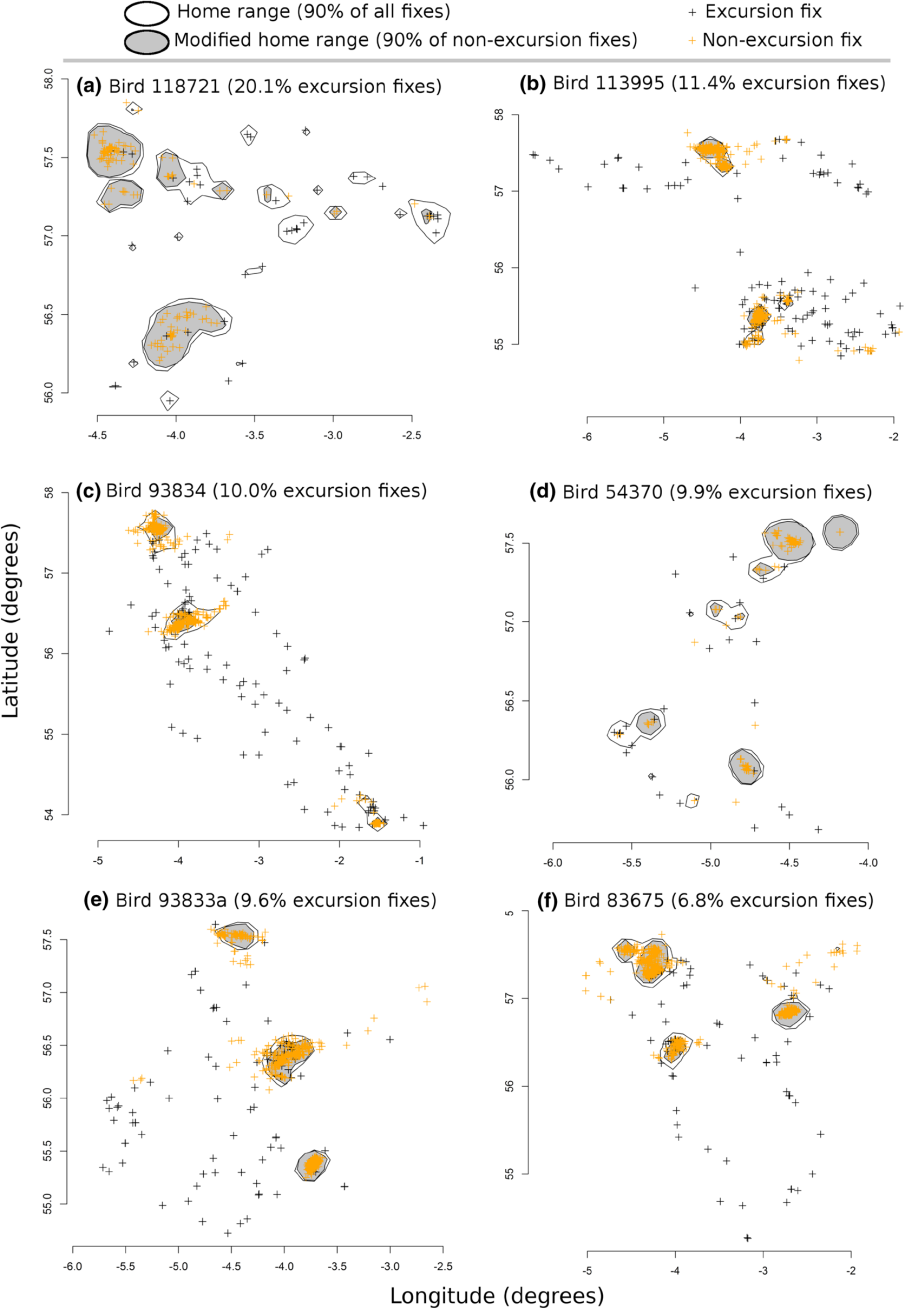
Visual representation of home ranges and location fixes of six individual red kites for which the greatest percentage of all location fixes was identified as being part of excursions (% given next to bird ID—see Table 1 for further information). *Black crosses* indicate ‘excursion fixes’, i.e. points on a movement path of high radial velocity and low angular velocity. *Amber crosses* indicate points that were not identified as being part of such excursion trajectories. The home ranges calculated after exclusion of all excursion fixes are presented in *grey* and overlay those calculated on 90 % of all location fixes (*white polygons*)

A total of 1237 fixes were identified as belonging to an excursion (Table 1), which was 5 % of all fixes (25 078). However, there was a considerable inter-individual variation in the prevalence of excursions. For example, over 20 % of all fixes were identified as belonging to excursions for one bird and around 10 % for a further four, while values were 0–7 % for the remaining 19 kites (Table 1). Graphical inspection indicated that the two-step process of identifying excursions was effective, with the majority of clearly outlying fixes identified as ‘excursion fixes’ (Fig. 4), and the majority of the remaining fixes identified as being part of clusters (i.e. temporary settlement areas); the latter means that the algorithm correctly identified the typically small and constricted movement within a home range (Fig. 2a). Direct movements between separate areas of intense use (Fig. 2b) were also correctly identified; for example, kite 83675 made regular flights between two well-separated parts of its home range (i.e. on either side of a city) and the algorithm identified such movement as excursion fixes (see Fig. 4f, top left ‘figure of eight’ polygon). Travel between greatly distant settlement areas was identified correctly (e.g. Fig. 4c, with identified travel between clusters as far apart as Inverness, Stirling and Leeds), as were excursions in various directions to places that may in the future become new settlement areas if the current pattern of frequenting were to continue (Fig. 4b, lower part). Finally, round trip excursions (movement starting and ending in the same temporary settlement area; Fig. 2c) were identified accordingly (Fig. 4f), indicating that, despite their simplicity, the algorithms were successful in identifying the variety of journeys exhibited among our sample of red kites.

### Calculation of home ranges

**Fig. 5:**
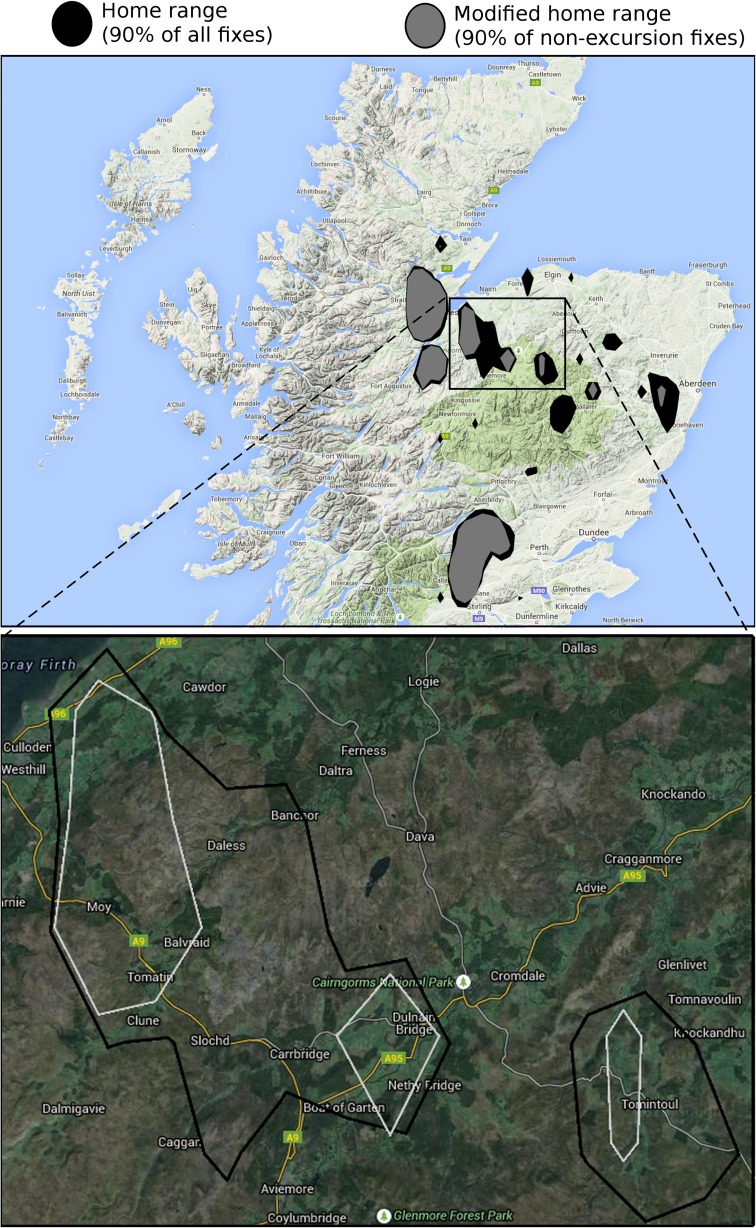
Temporary settlement areas (using 90 % kernels) of red kite 118731 overlaid on Google Maps, before (*black polygons*) and after (*amber polygons*) exclusion of all excursion fixes (20 % of all fixes): **a** All settlement areas across Scotland overlaid on Google Maps terrain layer; and **b** two settlement areas overlaid on Google Maps satellite view, showing ecologically superior delineation when excursion fixes were first removed (i.e. exclusion of moorland and mountain areas (*brown*) and inclusion of grassland (*light green*) and surrounding woodland (*dark green*)

The exclusion of excursion fixes generally led to the identification of ‘tighter’ clusters (i.e. a more refined estimate of the spatial extent of a temporary settlement area); in one case (118721, the bird with the highest percentage of identified excursion fixes; Fig. 4a), this prevented the inclusion of a spurious settlement. Whether such tighter delineations were generally ecologically superior descriptors of ‘areas used’ would require further investigation, but in some cases this appeared to be the case (see Fig. 5 for the correct exclusion of mountain areas from a kite’s home range). In other cases, no difference was discerned between the two clustering approaches, notably occurring when a kite had remained largely sedentary (i.e. few excursion fixes identified).

### Communicating ecological interpretations

**Fig. 6:**
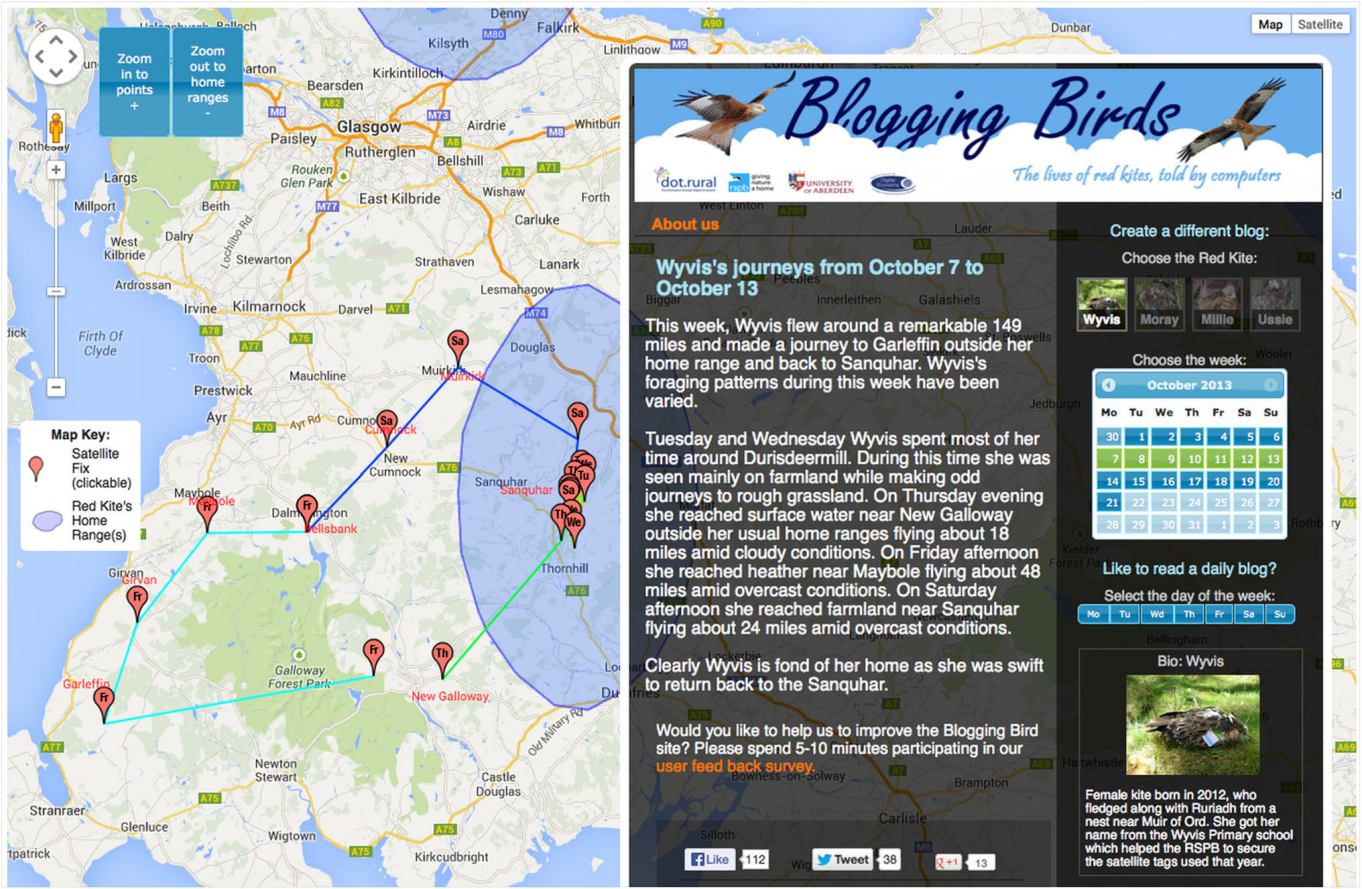
Screenshot of Blogging Birds (http://redkite.abdn.ac.uk), the website used to communicate the movement of four satellite-tagged red kites to a broad audience. The website provided weekly updates on journeys made by the focal red kites using Natural Language Generation technology (Ponnamperuma et al. 2013). Shown here are data of kite Wyvis (bird ID 93833a) for the week of 7 to 13 October 2013. The Google map portrays the latest calculated home ranges (*blue ovals* on 90 % of all non-travel fixes) and the *red markers* (with an abbreviation of the day of the week therein) show the actual GPS locations of this week which collectively reveal a three-day round trip). The identification of historical home ranges and travelling trajectories allowed for the real-time interpretation of this kites’ behaviour here for example leading to the text: “made a journey to Garleffin outside her home range and back”. Reference is also made to weather conditions, place names and terrain information, by linking location fixes to open data from the Met Office and Ordnance Survey

The automated identification and exclusion of excursions and the subsequent automated identification of home ranges (on the remaining ‘non-excursion’ fixes) allowed us to communicate the resulting ecological interpretations in real time to a wide variety of audiences (aim 3). To do this, we incorporated excursion and clustering routines into a system called ‘Tag2Blog’ (Ponnamperuma et al. 2013), resulting in the Blogging Birds website.^4^ This platform allowed us to bring four individual red kites ‘to life’ through short blogs (one for each satellite-tagged kite) automatically generated by Natural Language Generation software and based upon our automated interpretations of the movements of individual red kites (Fig. 6). The information on whether an individual kite was within its home range or undertaking an excursion was central to communicating the habits of red kites, although the blogs were further enriched with data from a wide variety of additional sources (e.g. meteorological and ordnance survey data; Ponnamperuma et al. 2013). As additional movement data were continuously captured by the satellite tags, the blogs were continuously updated when called up by a user. Home ranges were re-calculated on a weekly basis and new fixes compared with these and previous analyses to provide reference to the use of particular temporary settlement areas. This resulted in sentences such as: “This week, Moray was actively exploring a large area mainly within her home range”, or “This week Moray made a journey to Garleffin outside her home range and back” (see Box 1 for further quotes). Thus, the movement algorithms allowed us to continuously interpret and communicate, through daily and weekly blogs, animal movement based on satellite-tag data, specifically capturing whether a focal kite: a) remained within a home range, moved between different parts of a home range, or established a new home range; b) travelled between known home ranges; or c) explored areas outside known home ranges.

**Box 1:** Brief extracts from weekly blogs (http://redkite.abdn.ac.uk) which were automatically generated by a computer and built on real-time analysis of GPS satellite-tag data. Respective quotes are listed under headings which describe the different kinds of movement (see also Fig. 2) identified across our sample of red kites

Remaining within a home range:
“This week, Moray was actively exploring a large area mainly within her home range”. (8–14 July 2013)
“Millie’s foraging patterns during this week have been varied and Millie roosted largely in woodlands around Dingwall and Strone”. (1–7 April 2013)
“This week Millie was active. She predominantly flew between Errogie and Bunkegivie”. (18–14 March 2013)
Establishing a new home range:
“This week, Ussie was exploring a small area within his newly established home range”. (19–25 August 2013)
Travelling between known home ranges:
“Millie had enough of the area around Errogie and decided to move to her other home range around Nairn about 24 miles away. She is 2 years old and reaching her breeding age. Millie might be considering options for places to breed perhaps early as next year”. (25–31 August 2014)
Round trip:
“This week, Moray flew around a remarkable 115 miles and made a journey to Dingwall outside her home range and back to Torness”. (27 May–2 June 2013)
Exploring outside known home ranges:
“This week, Millie was exploring a small area outside her home range. Millie has been observed feeding mainly on heather during this week while roosting largely on woodlands around Nairn”. (11–17 August 2014)

## DISCUSSION

Our study demonstrates how a relatively simple algorithm—a combination of radial and angular velocity—can allow for real-time interpretation of satellite-tag data, which in turn permits immediate visualisation and communication of the resulting ecological insights for a wide range of purposes and audiences. The principle of using speed and angles to categorise satellite fixes is not new. For example, speed between successive locations and angles created by three consecutive locations, together with the GPS manufacturer’s quality index and number of satellites used for location calculation, was used to identify and remove locations with high error to enable fine-scaled spatio-temporal patterns of movement by loggerhead turtles (*Caretta caretta*), which also led to more accurate home-range estimates (Shimada et al. 2012). Morales et al. (2004) proposed an actual framework for fitting multiple random walks to animal movement paths consisting of ordered sets of step lengths and turning angles. Yet others developed equally advanced routines to detect and remove noise (e.g. the use of a Bayesian framework to provide location estimates with built-in measures of uncertainty; Sumner et al. 2009), as well as to identify animal behaviour [e.g. hidden Markov models to resolve spatial use aided by supplementary environmental data (Pedersen et al. 2011), or switching state-space models to readily identify distinct classes of movement behaviour (Jonsen et al. 2007)]. Unlike those models, our approach is remarkably simple; we show that speed of movement and turning angle derived from relatively sparse data allows for effective categorisation of location fixes, and rather than using it to reduce measurement error we employed algorithms to help identify a type of behaviour different from that expressed when occupying a home range or temporary settlement area.

The determination of home ranges, and thereby the handling of ‘outlying location fixes’ has a long history (Kie et al. 2010). While some studies may have manually removed fixes that did not fit the typically short-distant pattern of movement associated with home-range use, and others developed a family of Bayesian state-transition models to detect different movement behaviours from GPS data (e.g. Morales et al. 2004; Jonsen et al. 2007), it is somewhat remarkable that such an approach does not seem implemented as a default even in situations where excursions are extensive. For example, ringed seals (*Phoca hispida*) in the study of Kelly et al. (2010) ranged up to 1800 km from their home ranges but the authors did not remove such excursion data prior to home-range calculations. Likewise, a study on spiny dogfish (*Squalus acanthias*) did not remove fixes that formed part of distinct excursion behaviour (Carlson et al. 2014). In the latter study, however, the excursion points were effectively excluded by the kernel approach used as all were identified as ‘most outlying’. Indeed, if a relatively small percentage of all fixes were excursions (and thus almost by definition outlying), then approaches which use somewhat less than 100 % of all fixes for home-range calculation will suffice. Our algorithm identified 5 % of fixes across all our tagged individuals as belonging to excursions; hence a 95 % kernel approach may be expected to exclude many of those, meaning that from a home-range calculation point of view, there is little benefit to identify and remove excursion fixes first. However, for five of the 24 kites 10–20 % of the location fixes were identified as belonging to journeys. In these cases, a priori removal of travel fixes led to tighter home ranges, though the improvement was considerable for two birds only (Fig. 4a, d); its biological significance, however, remains to be investigated. Our investigation revealed that post-fledging dispersal for this species of raptor concerns the use of different settlement areas and—for most individuals—relatively little movement out with those. Part of this may be due to the existence of roosts used by primarily young birds (Heptinstall, unpublished), or to artificial or other long-term and predictable feeding opportunities (such as the known kite feeding station at 56.208°N/-4.038°E which was heavily frequented by four of the birds in Fig. 4a, c, e, f) used by kites well after having given up that area for temporary settlement. Had we applied our approach to a less site-loyal species, then considerably greater discrepancy in home ranges with and without excursion fixes should be expected. Further unravelling the ecological circumstances that led to the temporal settlement areas and travelling observed, as well as testing our routines on species with different movement behaviour are two subsequent steps that need to be taken.

While one of the benefits of identifying excursion fixes is that they can be removed prior to home-range calculations, another key benefit is that it allows for instantaneous interpretation of movement behaviour. Indeed, for our focal species, the latter proved most important as this enabled us to reveal journeys in a very early stage and communicate this through a dedicated website which allowed practitioners and local community members (as well as wider members of the public) to follow the movements of a small number of reintroduced red kites. We chose our threshold of radial and angular velocity (and several other heuristics) early on and thus relatively arbitrarily (but see Fig. 10.1007/s13280-015-0711-3 for their overall distributions). Ideally, such thresholds are set on the basis of firm ecological understanding, and the selection thereof could be automated in itself (to capture variation within a species if required). Once excursions are identified, several other statistics describing animal movement can be readily calculated. So far, we only produced estimates for distance travelled in certain time periods. Had we worked with other study systems, such as seabirds, then foraging range and trip duration would be meaningful candidate descriptors. Each descriptor may have its own challenges but once overcome, the use of simple automated algorithms to rapidly disclose ecological insights is likely to be rewarding, and recommendable particularly for projects where long-term movement data are being collected routinely.

Our algorithm to identify excursions was part of a larger undertaking aimed at automating the portrayal of satellite-tag data on maps and accompanying those with blogs written by a computer which capture the movement of several reintroduced red kites (Fig. 5). Although location data of the satellite-tagged kites had been available for many years, it was only since the launch of our website that these data were available in an accessible form to conservation officers responsible for the red kite reintroduction programme on the Black Isle. Thus, our technology helped conservation practitioners to better understand the lives of red kites and particularly how they re-colonised a landscape that held precious few kites for well over a century. To further assist nature conservation, a functionality similar to ‘Blogging Birds’ has now been worked up to portray spatial movement of satellite-tagged golden eagles (*Aquila chrysaetos*), a species of great cultural and conservation importance to Scotland, to allow members of the public to better understand how this charismatic species uses the Highlands. Given that persecution of raptors in the UK is on-going, initial concern was expressed by conservation officers regarding the risk of disclosing potentially sensitive locations, such as nest sites and roosts. We therefore provided a functionality to suppress the plotting of individual kite location data points but this was never used, partially because over time benefits associated with wide dissemination were deemed more important. For eagles, which arguably are even more sensitive, the plotting of mid-day fixes only was deemed to effectively mitigate any heightened risk associated with automated display of location data.

While the automated blogging site is highly advanced, it was the simple algorithm identifying whether a bird was likely on excursion or not that formed the basis of real-time communication of ecological insights. We recommend that such simple algorithms are used in satellite-tag studies to discriminate between home range and excursion behaviours, as this opens doors for rapid disclosure of insights to the benefit of the study and conservation of wildlife.

## Electronic supplementary material

Supplementary material 1 (PDF 553 kb)

## Footnotes

1 http://www.snh.gov.uk/about-scotlands-nature/species/fish/sea-fish/shark-tagging-project/.

2 e.g. http://www.bto.org/science/migration/tracking-studies/cuckoo-tracking.

3 http://www.microwavetelemetry.com/bird/solarArgosGPS_22g.cfm.

4 http://redkite.abdn.ac.uk.
