# Supplementary material for: Automated data analysis to rapidly derive and communicate ecological insights from satellite-tag data: A case study of reintroduced red kites
Source: Ambio. 2015 Oct 27;44(Suppl 4):612–23. doi: 10.1007/s13280-015-0711-3 (PMC4623868; doi:10.1007/s13280-015-0711-3)
Supplement: Supplementary file 1 — Supplementary material 1 (PDF 553 kb) [file 13280_2015_711_MOESM1_ESM.pdf]

Ambio

Electronic supplementary material

Title: **Automated data analysis to rapidly derive and communicate ecological insights from satellite-tag data: A case study of reintroduced red kites**

Authors: René van der Wal, Cheng Zeng, Danny Heptinstall, Kapila Ponnamperuma, Chris Mellish, Stuart Ben, Advaith Siddharthan

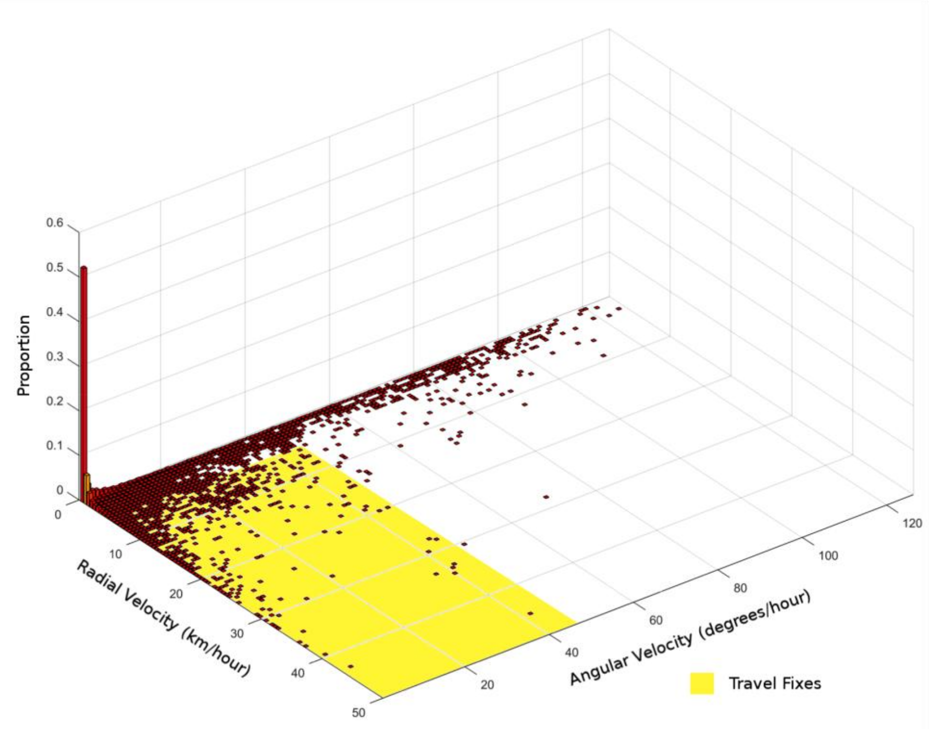

Fig. S1 Frequency distribution of angular and radial velocity data across our sample of red kite satellite-tag data. The area in yellow delineates conditions in which angular velocity is sufficiently low (< 45°/hr), and radial velocity sufficiently high (> 5km/hr), for location fixes to be categorised as 'excursion fixes'. The data were calculated such that all 24 kites contributed equally to the proportions plotted.
